# Virulence of *Metarhizium robertsii* Strains Isolated from Forest Ecosystems Against Wax Moths (*Galleria mellonella*, *Achroia grisella*) and Pine Processionary (*Thaumetopoea pityocampa*) Larvae

**DOI:** 10.3390/biology14081009

**Published:** 2025-08-06

**Authors:** Spiridon Mantzoukas, Vasileios Papantzikos, Chrysanthi Zarmakoupi, Panagiotis A. Eliopoulos, Ioannis Lagogiannis, George Patakioutas

**Affiliations:** 1 Institute of Mediterranean Forest Ecosystems, 115 28 Athina, Greece; 2 Department of Agriculture, University of Ioannina, Arta Campus, 471 00 Arta, Greece; b.papantzikos@uoi.gr (V.P.); chris.zarm@hotmail.com (C.Z.); gpatakiu@uoi.gr (G.P.); 3 Laboratory of Plant Health Management, Department of Agrotechnology, University of Thessaly, 415 00 Larissa, Greece; eliopoulos@uth.gr; 4 ELGO-Demeter, Plant Protection Division of Patras, NEO & L. Amerikis, 264 44 Patras, Greece; lagoipp@gmail.com

**Keywords:** entomopathogenic fungi, virulence, moth larvae, lepidoptera, *Metarhizium robertsii*, forest ecosystems, larvae

## Abstract

Entomopathogenic fungi (EPF) are one of the most environmentally friendly ways to control a plethora of chewing insects such as *T. pityocampa*, *G. mellonella*, and *A. grisella*. Bioassay of EPF on these highly damaging pests is considered important in the face of climate change in order to research alternative solutions that are capable of limiting chemical control, the overuse of which increases insects’ resistance to chemical compounds. In this study, the insecticidal virulence of *Metarhizium robertsii* isolates, retrieved from forest ecosystems, was tested on second-instar larvae of *T. pityocampa*, *G. mellonella*, and *A. grisella*. Bioassays were carried out in the laboratory, where experimental larvae were sprayed with 2 mL of a six-conidial suspension from each isolate. Mortality was recorded for 144 h after exposure. Mean mortality, lethal concentrations, sporulation percentage, and sporulation time were estimated for each isolate. *Metarhizium* isolates resulted in the highest mortality (89.2% for *G. mellonella* and 90.2% for *A. grisella*). Based on the LC_50_ estimates determined by the concentration–mortality relationships for the tested fungal isolates, we demonstrated significant virulence on larvae of *G. mellonella*, *A. grisella*, and *T. pityocampa*. Our results indicate that entomopathogenic fungi have the potential to become a very useful tool in reducing chemical applications.

## Simple Summary

Moth pest populations often thrive under rising temperatures, an increasingly challenging consequence of climate change that results in the expansion of their populations to new areas and hosts. In recent years, pine forests in several countries have been at great risk due to the significant spread of the pine processionary moth *Thaumetopoea pityocampa* (Lepidoptera: Thaumatopoeidae). In addition, *Galleria mellonella* (Lepidoptera: Pyralidae) and *Achroia grisella* (Lepidoptera: Pyralidae) are the major pests of beehives. Chemical treatment of these moths is often suboptimal, on the one hand due to the large and protected habitats involved, such as forests in the case of *T. pityocampa*. On the other hand, in the case of *A. grisella*, where bees are concerned, the management must be carried out in a non-invasive chemical way that may only partially reduce pest populations.

## 1. Introduction

The processionary moth *Thaumetopoea pityocampa* is one of the main pine tree defoliators, threatening the pine forests of many Mediterranean regions in Eastern Europe and Western Asia [1]. It builds silk nests around the pine branches [2] and creates significant ecological and economic damage to many Pinus species, such as *Pinus nigra* L. *Pinus halepensis* L., and *Pinus brutia* L. (Pinales: Pinaceae) [3,4]. The most damaging stage of *T. pityocampa* is the larvae [5] cutting pine needles, causing around 60% of losses to pine trees [6] and making them susceptible to secondary infestations by ambrosia beetles (Coleoptera: Scolitynae) [2]. Scots pine *Pinus silvestris* L. (Pinales: Pinaceae) seriously suffers from the feeding damage of *T. pityocampa*, which develops quite well, consuming pine needles [3,7] according to laboratory tests [4].

*Achroia grisella*, commonly known as the lesser wax moth, is a significant pest in honey production [8]. It is usually referred to in the literature as a secondary pest for agriculture, and this may be a reason for its low occurrence in research [9]. *A. grisella* is spreading by high-altitude and -latitude distribution [10]. Its presence increases significantly within abandoned beehives [11]. Adult females are very active at night, flying around beehives and laying their eggs on honey wax combs. Larvae and pre-pupae stages are usually detected in depressions in the wooden walls of the beehives [11], and they begin to feed after a few days, destroying combs by boring through the cells to feed with cast skins and pollen. To stay safe, they spin silk galleries in the beehive as they feed on wax, leaving only dusty debris behind [11]. Argentina used to be the second highest natural honey exporter, and the presence of beehive pests such as *A. grisella* may be associated with the significant economic drop-down in its production recently, along with other factors [12]. Given the impact of moth pests’ behavior on bees, *A. grisella* control is critical, because it may unfavorably impact bee population levels.

The greater wax moth, *Galleria mellonella* (Lepidoptera: Pyralidae), is a cosmopolitan pest that causes significant damage to bees [13]. It is a holometabolous insect with four developmental stages [9,14] that can be found in beehives or stored waxes. It destroys the beehives by feeding on them, causing a phenomenon called galleriosis [15]. It spins silk tunnels when consuming large amounts of beeswax, honey, pollen residues, or exuviae of bee larvae [16], making holes through which honey leaks out [17,18]. Larvae are the most consuming stage, and within a week of colonization, they can totally destroy the beehive [19], leading to significant damage to the honeycombs [20]. Furthermore, *G. mellonella* is responsible for the transmission of the black queen cell virus and the Israeli acute paralysis virus, which potentially endangers honeybees [17,18]. This damage was very high in Florida (USD 3 per colony) and Texas (USD 1.5 per colony) in 1997 [21].

Treatment of *T. pityocampa*, *A. grisella*, and *G. mellonella* is crucial because of the serious damage they cause, which is strongly connected to their climatic adaptation [10,22,23]. They can cope with rising temperatures [10,24] and reproduce rapidly inside their nests, leading to hundreds of individuals [5,23,25], and based on these evolutionary advantages, they can progressively expand their populations. Chemical control of *A. grisella* has been attempted using para-dichlorobenzene and naphthalene [26]. Chemical fumigants such as acetic acid, methyl bromide, paradichlorobenzene (PDB), calcium cyanide, ethylene bromide, sulfur, phosphine, and carbon dioxide are usually implemented for *G. mellonella* treatment in beehives [27]. However, the aforementioned compounds, except carbon dioxide, pose quality risks on beehive products such as honey due to chemical residues [28] and are not lethal for *G. mellonella* eggs, except PDB [18]. Moreover, these compounds have a negative impact on the environment, are not always considered suitable for their broad application in forests [29,30], and have adverse effects on the host plants [31] or beehives [32,33]. Therefore, an alternative approach with safe biological agents is required. Moreover, the eco-trap strategy for *T. pityocampa* is not always an affordable method of control when used in large pine forests [34,35]. 

Entomopathogenic fungi (EPF), *Beauveria bassiana* (Hypocreales: Cordycipitaceae), and *Metarhizium anisopliae* (Hypocreales: Clavicipitaceae) have been successfully used against lesser wax moth’s larval and pupal stages [36]. The same EPF have been efficient against *T. pityocampa*, causing 100% mortality to third-instar larvae ten days after application [37]. *B. bassiana* and *Paecilomyces lilacinus* (Hypocreales: Ophiocordycipitaceae) can cause high larval mortality to *G. mellonella* [38]. Additionally, *Metarhizium bruneum* (Hypocreales: Clavicipitaceae) has been successful against most larval instars of *T. pityocampa* [39], as well as their eggs [35]. Biocontrol using EPF is one of the most promising insect control strategies and could be safely used in integrated pest management programs (IPMs) [40], especially in forest ecosystems [41], presenting no side effects for bee populations [42,43,44].

*T. pityocampa*, *A. grisella*, and *G. mellonella* inflict serious ecological and economic damage, and under the pressure of climate change, it is necessary to focus on ecologically friendly methods to reduce their damage to beehives. The present study aimed to evaluate the insecticidal activity of *Metarhizium robertsii* (Hypocreales: Clavicipitaceae) on *T. pityocampa*, *A. grisella*, and *G. mellonella* as an environmentally viable alternative to chemical insecticides.

## 2. Materials and Methods

### 2.1. Fungal Strains

The two strains used in this study were *M. robertsii* (=MET S) (20140422CS3P4_C02_2016-04-27), which was isolated from *G. mellonella* (Paphos Forest, Cyprus, 35.064236), and *M. robertsii* (=MET K) (Blast ID D9JJ45D9301), which was isolated from *Sitophilus granarius* (Coleoptera: Curculionidae) (Rouva Forest Crete, Greece, 35.180514, 24.903244). The viability of all the tested fungi was determined by spreading a 100 μL aliquot of a conidia suspension (1 × 10^6^ conidia/mL), prepared with a sterile surfactant solution (0.05% v/v) of Tween 80, on Sabouraud dextrose agar (SDA) in Petri dishes (90 × 15 mm) and incubating it in the dark at 25 ± 1 °C. SDA plates of the tested fungi were incubated for 18 h prior to evaluation. Conidia were scored as viable if any germ tube was 2× longer than the diameter of the spore, with a total of 100 conidia per sample under 400× magnification. Conidial viability was calculated based on the formula below:

Viability (%) = [G1/(G1 + G2)] × 100

where G1 refers to the number of germinated conidia, G2 is the number of non-germinated conidia, while the sum of G1 and G2 is equal to 100. Thus, the viable conidia percentage was determined by counting a total of 100 conidia per fungal sample. Fungal strains presenting ≥95% viability were used in the insect bioassays. Conidia had >97% viability. 

### 2.2. Insect Rearing in the Lab

*G. mellonella*, *A. grisella*, and *T. pityocampa* larvae were reared and incubated in the insectarium of the University of Ioannina, Department of Agriculture, and at Institute of Mediterranean Forest Ecosystems, Entomology Lab, under controlled conditions of 26 °C and 70% relative humidity. *G. mellonella* and *A. grisella* larvae were initially collected from local apiaries in Arta (Greece) and *T. pityocampa* larvae from naturally infested pine trees (*Pinus halepensis*) in the same region. The artificial diets for *G. mellonella* and *A. grisella* larvae were placed in a sterilized beaker, where 58.3 g (22%) of glycerol, 58.3 g (22%) of organic honey, and 10 mL (4%) of water were combined. Then, the mixture was heated in a microwave at 1000 W for 1 min and allowed to cool to room temperature. Then, 250 g (48%) of cereal was mixed with the liquid until the mixture crusted; then, 8 g (4%) of instant dry baker’s yeast was added and mixed thoroughly. The culture medium was prepared fresh and not stored, as storing it caused the mixture to dry out. *T. pityocampa* larvae were supplied with fresh maritime pine needles, the primary host species in the pine forest. Fresh twigs were provided every one or two days. 

### 2.3. Laboratory Bioassay

**Figure 1:**
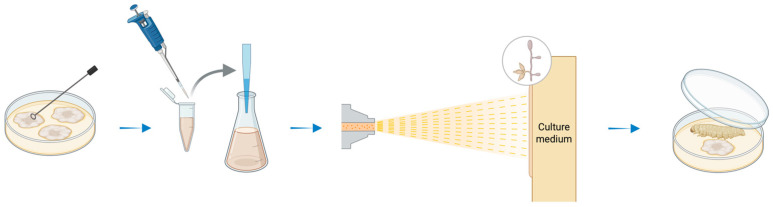
Depiction of EPF isolation and spraying on the culture medium.

Conidial suspensions of the *M. robertsii* were prepared at 10^3^,10^4^,10^5^, 10^6^, 10^7^, and 10^8^ conidia/mL to assess their insecticidal potential. One hundred 2nd-instar larvae were used for tests. Ten larvae were sprayed with 2 mL of EPF conidial suspension and then placed in 9 cm sterile Petri dishes (Figure 1). The prepared suspensions were applied at 1 kgf cm^−2^ using a Potter spray tower (Burkard Manufacturing Co., Ltd., Rickmansworth, Hertfordshire, UK). Treated larvae were maintained under the same rearing conditions and supplied with the artificial diet. Larval mortality was recorded daily for a seven-day experimental period. Ten Petri dishes (repetitions) were used per concentration, with ten (10) larvae each (6 concentrations × 10 replicates × 3 species = 1800 larvae in total). Untreated larvae sprayed only with 10 mL of surfactant solution (Tween 80, 0.05%) were used as a control. Dead larvae were removed and surface-sterilized with 2% sodium hypochlorite for a few seconds to avoid the development of saprophytic fungi. Following this, they were transferred and kept in the dark at 25 °C for 5–7 days, and those that showed fungal growth were classified as infected. EPF species from each dead larva were initially identified under a microscope via observation of the conidia and the hyphal growth.

### 2.4. Statistical Methodology

Corrected mortality percentages were calculated using Abbott’s formula and arcsine-transformed before analysis. Data were then analyzed by means of univariate ANOVA involving a multi-factor analysis, using the general linear model of the SPSS (version 26). In case of significant *F* values, means were compared using the Bonferroni test. LC_50_ values were calculated by probit analysis with a 95% confidence interval (CI). The percentages of sporulating cadavers and the median sporulation time were compared using one-way ANOVA to determine differences between isolates. 

## 3. Results

Significant differences were recorded between the insect, fungal isolate, dose, and the day of the experiment factors in relation to the dependent variable of mortality (Table 1). The effectiveness of the *Metarhizium* isolates was significant against the second-instar larvae of *G. mellonella*, *A. grisella*, and *T. pityocampa* in different. The four-way factor model of Insect * Days * Dose, Insect * Fungal Isolates * days, and Fungal Isolates * Dose * Days and the four-way factor model of Insect * Fungal Isolates * Doses * Days also showed significant effects in terms of the mortality of larvae at 144 h.

The mortality percentage depended on the fungal isolate, tested dose, and larvae sensitivity to the fungal isolate. The final mortality percentages of *G. mellonella* larvae after 144 h ranged from 28.4 to 87.8% in the treatments with Met S isolate and 27.7 to 89.2% in the treatments with Met K isolate (Table 2). The final mortality percentages of *A. grisella* larvae were 26.4 to 88.3% in the treatments with Met S isolate and 29.2 to 90.2% in the treatments with Met K isolate (Table 3). 

The final mortality percentages of *T. pityocampa* larvae were 29.4 to 89.8% in the treatments with Met S isolate and 28.9 to 88.1% in the treatments with Met K isolate (Table 4). For control larvae who had been treated only with H_2_O + Tween 80, the mortality was 3.3% for the *G. mellonella* larvae, 1.7% for the *A. grisella* larvae, and 3.3% for the *T. pityocampa* larvae at the end of the experiment (Table 2, Table 3 and Table 4).

Probit analysis results revealed that the median lethal concentrations (LC_50_) of Metarhizium isolates (Met S and Met K) from *G. mellonella* larvae were estimated to be 9.24 × 10^4^ conidia/mL (for Met S) to 9.12 × 10^4^ conidia/mL (for Met K), while from *A. grisella* larvae, they were estimated as 7.82 × 10^4^ conidia/mL (for Met S) to 6.99 × 10^4^ conidia/mL (for Met K), and finally, for the *T. pityocampa* larvae, they were estimated to be 5.10 × 10^4^ conidia/mL (for Met S) to 5.31 × 10^4^ conidia/mL (for Met K) (Table 5).

A high rate of mycosis was observed on cadavers of second-instar larvae of *G. mellonella*, *A. grisella*, and *T. pityocampa* for Met S [73.3% (*G. mellonella*) and 71.7% (*A. grisella*)] and for Met K [69.8% (*T. pityocampa*) and 69.2% (*G. mellonella)*] (F = 12.144; df = 2; *p* = 0.213) (Table 6). Moreover, the shortest sporulation time was recorded in cadavers treated with Met S (3.93 days) for the *G. mellonella* as well as *A. grisella* larvae (4.11 days) (F = 10.178; df = 2; *p* = 0.189) (Table 6). 

## 4. Discussion

The insecticidal efficacy of an EPF is significantly impacted by many factors, such as insect behavior, population density, age, nutrition, and genetic information [45,46]. The mode of entry of entomopathogenic fungi is generally by contact, while *M. robertsii* is also capable of penetrating through the insect cuticle, producing hydrolytic enzymes, i.e., proteinases, chitinases, and lipases, which enable infection against many Lepidoptera [47]. The pathogenicity of fungi relies on their ability to overcome innate immunity, and in the case of EPF, this involves the secretion of proteinases that breach the cuticle and allow for colonization of the insect host. EPF have been used to control insect pests, and they play an important role in the regulation of insect populations in nature [45,47].

This study reports that the *M. robertsii* strains Met S and Met K were highly pathogenic to second-instar larvae of *G. mellonella*, *A. grisella*, and *T. pityocampa*. Older instars are generally more resistant to pathogens, likely due to their thicker cuticles and behavioral differences. Additionally, they possess urticating setae that may trap conidia, partly explaining their increased resistance. This may be attributed to larval behavior and differences in cuticle structure. First- and second-instar larvae may not tend to crawl over each other as much as the older instars. The time between ecdysis may also be shorter than for the older instars. Furthermore, older instars possess urticating setae, which could trap conidia; this may partly explain their greater resistance. *A. grisella* is the least studied of the three tested lepidopterans as far as myco-biological control is concerned. 

In the present study, both *M. robertsii* isolates demonstrated high pathogenicity against second-instar larvae of *G. mellonella*, *A. grisella*, and *T. pityocampa*. Our results are consistent with those of Seyoum and Namusana, 2010 [48], where over 85% mortality of *A. grisella* larvae could be achieved by day 8 post-treatment with most fungal isolates, and with that of Girişgin et al. 2022 [49], who reported significant suppression (88.35%) of larvae of *A. grisella* by commercial *M. anisopliae* formulations. Similarly, Sönmez et al. 2017 [39] mentioned that the application of different concentrations of *M. brunneum* isolates resulted in 46.7–100% and 60–100% mortality against fourth-instar larvae of *T. pityocampa*. Similar results have been reported by Er et al., 2007 [50], in whose study three isolates of *P. fumosoroseus*, one isolate of *B. bassiana*, and one isolate of *M. anisopliae* caused almost complete mortality to fourth-instar larvae of *T. pityocampa*. Studies showed that *Metarhizium* species had a high virulence against the third larval instar of *T. absoluta* (Lepidoptera: Galechiidae) [51,52]. Wakil et al. 2013 reported that *B. bassiana* and *Beauveria brongniartii* (Lepidoptera: Cordycipitaceae) were the most pathogenic fungal species against the larvae of wax moth *G. mellonella* compared with the other fungi [53]. High larval mortalities have also been recorded (96.5 and 89.6%) when *G. mellonella* was treated with conidial suspension of *B. bassiana* strains [54].

The insecticidal efficacy of EPF is highly influenced by several other factors, such as the insect’s behavior, population density, age, nutrition, and genetic information; environmental conditions; as well as the effect of host physiology and morphology on its sensitivity to biological control agents such as EPF [55]. Therefore, the differences in insects’ susceptibility to EPF could not be explained solely as a function of the applied conidial concentration [56]. The differences in experimental methodology, fungal isolate virulence, and weevil strain are the main factors that cause this great variation in recorded mortalities.

Based on the LC_50_ estimates determined by the concentration–mortality relationships for the tested fungal isolates, they had lethal effects on larvae of *G. mellonella*, *A. grisella*, and *T. pityocampa*. Thus, the two isolates of *M. robertsii* warrant further investigation for microbial control of lepidopteran larvae. Perhaps more attention should be given to *T. pityocampa* larvae, because the LC_50_ values were smaller in relation to the *G. mellonella* and *A. grisella* larvae. The results from the insect bioassays indicate that *G. mellonella*, *A. grisella*, and *T. pityocampa* are susceptible to infection by *M. robertsii*. However, comparing the LC_50_ values showed differences in the mortality rates, sporulation percent, and time at the second larval stages. Second-instar larvae of the tested Lepidoptera exhibited different sensitivities to fungal infection, highlighting the importance of knowing the target insect’s developmental stage for effective fungal application.

Some researchers mention that the variability in larval susceptibility is linked to molting, since shedding of the older cuticle results in the removal of the fungal inoculum [39]. Entomopathogenic fungi appear to have more specific requirements for germination [47]. In the case of the three tested lepidopteran larvae, the sporulation percent was above 70%, especially in the case of the Met S isolate. The number of mycoses cadavers suggested that mortality was likely EPF-induced, rather than stress-related. The sporulation times for Met S were 3.82 days (*T. pityocampa*), 3.93 days (*G. mellonella*), and 4.11 days (*A. grisella*). On the other hand, for the Met K isolate, the sporulation time was slightly higher for all tested larvae. It is well established that the germination speed of the conidium influences its virulence, and consequently, in general, conidia that germinate faster are more virulent to insects [57,58,59,60], suggesting that virulence depends significantly on the speed of infection.

## 5. Conclusions

Tested native isolates have shown promising results in the present laboratory-based experiments. The favorable conditions in the laboratory might have enhanced the EPFs’ performance, although this may not reflect the nature of field ecosystems, where suboptimal conditions for growth and viability, many different antagonists, and adverse weather conditions may prevail. The present study revealed the possibility of isolating the *M. robertsii* strains MET S and MET K from the local environment to manage *G. mellonella*, *A. grisella*, and *T. pityocampa*. Most of the *M. robertsii* strains, especially MET S and MET K, showed a high pathogenicity against second-instar larvae of *G. mellonella*, *A. grisella*, and *T. pityocampa*. The strains with high mortality, high sporulation, and lower sporulation times are promising for controlling the target pest at an early stage, as well as producing economical conidia for pest management. Further field evaluation of these strains is needed to determine their potential, especially for *T. pityocampa*.

## Figures and Tables

**Table 1:** ANOVA parameters for mortality levels of *G. mellonella*, *A. grisella*, and *T. pityocampa larvae* exposed for 144 h to six doses of *M. robertsii* under laboratory conditions.

Factor	df	F	Sig.
Insect	2	6.773	<0.001
Fungal Isolate	1	15.373	<0.001
Dose	5	5.678	<0.001
Days	6	9.312	<0.001
Insect * Fungal Isolate	2	7.306	<0.001
Insect * Dose	10	0.366	0.900
Insect * Days	12	2.717	0.004
Fungal Isolate * Dose	5	2.118	0.085
Fungal Isolate * Days	6	8.833	<0.001
Dose * Days	30	1.651	0.132
Insect * Fungal Isolate * Dose	4	0.450	0.976
Insect * Fungal Isolate * Days	24	3.659	<0.001
Insect * Dose * Days	60	1.307	<0.001
Fungal Isolate * Dose * Days	30	3.097	<0.001
Insect * Fungal Isolate * Dose * Days	60	0.956	<0.001

**Table 2:** Mean mortality (±SD) of *G. mellonella* larvae exposed to Met S and Met K isolates for 144 h. Means within the same column followed by the same letter are not significantly different (Bonferroni test, *p* < 0.05).

Treatment/Mortality	Concentration	24 h	48 h	72 h	96 h	120 h	144 h
MET S	10^3^	0.0 ± 0.0 a	0.0 ± 0.0 a	3.4 ± 1.6 b	5.3 ± 2.7 b	16.8 ± 2.4 b	28.4 ± 1.6 bc
	10^4^	0.0 ± 0.0 a	2.5 ± 2.4 b	6.9 ± 1.8 b	10.5 ± 2.1 b	23.5 ± 1.5 b	40.6 ± 2.8 d
	10^5^	0.0 ± 0.0 a	3.8 ± 1.3 b	14.0 ± 0.0 b	27.4 ± 1.6 bc	39.2 ± 1.2 d	50.9 ± 1.1 e
	10^6^	5.3 ± 3.2 b	10 ± 0.0 b	18.5 ± 2.5 b	33.4 ± 1.4 c	56.6 ± 2.6 e	67.8 ± 1.5 f
	10^7^	6.8 ± 1.4 b	12.4 ± 1.5 b	21.0 ± 0.0 b	41.5 ± 2.6 d	65.4 ± 2.3 f	78.2 ± 1.2 h
	10^8^	10.0 ± 0.0 b	16.3 ± 1.3 b	26.4 ± 2.1 c	53.9 ± 1.6 e	84.7 ± 1.4 g	87.8 ± 2.5 i
MET K	10^3^	0.0 ± 0.0 a	2.4 ± 1.6 b	4.4 ± 2.2 b	7.3 ± 2.5 b	18.3 ± 1.5 b	27.7 ± 2.1 bc
	10^4^	0.0 ± 0.0 a	4.5 ± 1.4 b	9.9 ± 1.1 b	17.5 ± 1.8 b	34.5 ± 2.3 d	40.9 ± 2.4 d
	10^5^	0.0 ± 0.0 a	7.8 ± 1.2 b	18.3 ± 2.3 b	25.2 ± 1.5 bc	49.2 ± 2.2 e	55.3 ± 2.1e
	10^6^	7.4 ± 2.1 b	15.3 ± 2.3 b	22.5 ± 2.1 bc	33.4 ± 2.1 c	52.6 ± 1.2 e	69.3 ± 3.4 f
	10^7^	8.8 ± 1.8 b	18.4 ± 1.2 b	24.0 ± 0.0 c	40.3 ± 2.8 d	63.7 ± 1.5 f	75.6 ± 2.9 h
	10^8^	13.2 ± 2.4 b	19.2 ± 2.7 b	29.9 ± 2.1 c	50.9 ± 3.1 e	79.3 ± 1.5 h	89.2 ± 1.5 i
Control	H_2_O + Tween 80	0.0 ± 0.0 a	0.0 ± 0.0 a	0.0 ± 0.0 a	3.3 ± 2.3 b	3.3 ± 2.3 b	3.3 ± 2.3 b

**Table 3:** Mean mortality (±SD) of *A. grisella* larvae exposed to Met S and Met K isolates for 144 h. Means of the same column followed by the same letter are not significantly different (Bonferroni test, *p* < 0.05).

Treatment/Mortality	Concentration	24 h	48 h	72 h	96 h	120 h	144 h
MET S	10^3^	0.0 ± 0.0 a	2.9 ± 2.1 b	3.4 ± 1.5 b	5.3 ± 2.2 b	16.8 ± 1.8 bc	26.4 ± 1.6 c
	10^4^	0.0 ± 0.0 a	3.5 ± 1.5 b	6.6 ± 2.1 b	10.5 ± 1.9 b	23.5 ± 1.9 c	42.4 ± 1.2 e
	10^5^	0.0 ± 0.0 a	6.8 ± 1.2 b	13.4 ± 1.2 b	27.4 ± 1.6 cd	39.2 ± 3.1 e	56.3 ± 2.3 h
	10^6^	4.3 ± 1.1 b	10.0 ± 0.0 b	18.5 ± 1.3 c	37.4 ± 1.8 e	55.9 ± 0.9 h	67.8 ± 2.1 g
	10^7^	8.9 ± 1.7 b	12.4 ± 2.1 b	23.2 ± 1.2 c	43.5 ± 2.4 f	66.4 ± 1.1 g	73.2 ± 3.5 g
	10^8^	11.9 ± 2.2 b	17.3 ± 1.7 c	29.4 ± 1.6 d	64.9 ± 1.5 g	77.7 ± 2.1 i	88.3 ± 2.9 k
MET K	10^3^	0.0 ± 0.0 a	3.2 ± 1.4 b	4.4 ± 1.2 b	7.3 ± 2.7 a	15.2 ± 2.9 c	29.2 ± 1.8 d
	10^4^	0.0 ± 0.0 a	4.4 ± 1.3 b	9.9 ± 1.1 b	17.5 ± 2.5 c	34.5 ± 1.6 e	43.6 ± 1.4 e
	10^5^	0.0 ± 0.0 a	7.5 ± 2.1 b	18.3 ± 2.3 c	25.2 ± 1.8 d	49.2 ± 0.8 j	54.8 ± 1.1 h
	10^6^	7.3 ± 1.7 b	17.7 ± 3.7 c	22.5 ± 2.4 c	33.4 ± 1.6 e	52.6 ± 2.7 h	69.3 ± 2.1 g
	10^7^	7.8 ± 1.2 b	18.7 ± 2.2 c	25.8 ± 1.2 c	41.5 ± 0.9 f	63.7 ± 1.5 g	77.2 ± 3.2 i
	10^8^	13.2 ± 2.1 b	19.2 ± 2.7 c	29.9 ± 1.1 d	60.9 ± 2.1 g	78.3 ± 1.4 i	90.2 ± 1.8 k
Control	H_2_O + Tween 80	0.0 ± 0.0 a	0.0 ± 0.0 a	0.0 ± 0.0 a	0.0 ± 0.0 a	1.7 ± 2.3 b	1.7 ± 2.3 b

**Table 4:** Mean mortality (±SD) of *T. pityocampa* larvae exposed to Met S and Met K isolates for 144 h. Means within the same column followed by the same letter are not significantly different (Bonferroni test, *p* < 0.05).

Treatment/Mortality	Concentration	24 h	48 h	72 h	96 h	120 h	144 h
MET S	10^3^	0.0 ± 0.0 a	2.7 ± 1.3 b	5.4 ± 2.1 b	8.3 ± 1.7 b	18.9 ± 1.8 bc	29.4 ± 2.7 d
	10^4^	0.0 ± 0.0 a	3.5 ± 2.8 b	9.9 ± 1.6 b	13.5 ± 2.1 b	27.8 ± 1.5 d	42.6 ± 2.1 f
	10^5^	0.0 ± 0.0 a	5.8 ± 1.4 b	14.9 ± 2.1 b	27.4 ± 1.3 d	39.9 ± 1.9 f	56.7 ± 1.2 g
	10^6^	4.3 ± 1.9 b	10.9 ± 1.5 b	18.5 ± 1.4 bc	35.8 ± 1.8 e	56.6 ± 2.4 g	69.8 ± 1.9 i
	10^7^	8.9 ± 1.1 b	12.4 ± 0.9 b	24.7 ± 1.3 c	45.6 ± 2.9 f	68.4 ± 1.3 i	78.9 ± 0.9 j
	10^8^	13.6 ± 1.4 b	18.9 ± 1.1 bc	29.4 ± 1.1 d	57.9 ± 2.9 g	78.7 ± 1.9 j	89.8 ± 1.1 l
MET K	10^3^	0.0 ± 0.0 a	3.2 ± 3.3 b	4.4 ± 1.6 b	7.3 ± 1.7 b	16.8 ± 2.1 bc	28.9 ± 1.5 d
	10^4^	0.0 ± 0.0 a	4.4 ± 1.7 b	9.7 ± 2.3 b	17.5 ± 2.3 bc	34.5 ± 1.9 e	44.6 ± 1.8 f
	10^5^	0.0 ± 0.0 a	7.5 ± 1.3 b	18.3 ± 1.8 bc	25.2 ± 2.6 cd	49.2 ± 1.5 f	57.3 ± 2.6 g
	10^6^	6.3 ± 1.4 b	12.7 ± 1.4 b	21.6 ± 1.7 c	33.4 ± 1.9 e	52.8 ± 2.6 f	69.3 ± 0.9 i
	10^7^	6.5 ± 1.2 b	15.7 ± 1.6 b	23.2 ± 1.2 c	41.5 ± 2.1 f	63.7 ± 1.4 k	75.7 ± 2.2 j
	10^8^	11.4 ± 2.1 b	17.2 ± 1.8 bc	27.5 ± 0.8 d	50.9 ± 2.6 f	78.3 ± 2.1 j	88.1 ± 2.6 l
Control	H_2_O + Tween 80	0.0 ± 0.0 a	0.0 ± 0.0 a	0.0 ± 0.0 a	0.0 ± 0.0 a	3.3 ± 2.3 b	3.3 ± 2.3 b

**Table 5:** Estimated lethal concentrations for *Metarhizium* isolates on 2nd-instar larvae of *G. mellonella*, *A. grisella*, and *T. pityocampa*. **LCs**: lethal concentrations; **SE**: standard error; **CI**: confidence limit; **χ^2^:** Chi-squared goodness-of-fit test.

Insect	Fungal Isolate	df	LC^50^ (conidia/mL) 95% CL	Slope ± SE	Chi-test (χ^2^) Sig	Intercept
*G. mellonella*	Met S	4	9.24 × 10^5^ (8.80 × 10^4^–9.45 × 10^6^)	0.339 ± 0.51	0.997	3.380
	Met K	4	9.12 × 10^5^ (9.32 × 10^4^–8.95 × 10^6^)	0.341 ± 0.50	1.000	3.375
*A. grisella*	Met S	4	7.82 × 10^5^ (7.97 × 10^4^–9.11 × 10^6^)	0.342 ± 0.51	1.000	3.329
	Met K	4	6.99 × 10^4^ (7.16 × 10^3^–6.73 × 10^5^)	0.344 ± 0.50	1.000	3.334
*T. pityocampa*	Met S	4	5.10 × 10^4^ (5.26 × 10^3^–4.95 × 10^5^)	0.346 ± 0.53	1.000	3.373
	Met K	4	5.31 × 10^4^ (4.76 × 10^3^–5.93 × 10^5^)	0.322 ± 0.54	0.999	3.477

**Table 6:** The sporulation percentage and sporulation time on 2nd-instar larvae of *G. mellonella*, *A. grisella*, and *T. pityocampa*. Mean ± SD values with the same letter within a column are not significantly different (*p* < 0.05).

**Insect**	**Fungal Isolate**	**Sporulation on Cadavers (% + SD)**	**Sporulation Time on Cadavers (Days + SD)**
*G. mellonella*	Met S	73.3 ± 9.4 a	3.93 ± 0.2 a
	Met K	69.2 ± 8.7 a	4.17 ± 0.57 a
*A. grisella*	Met S	71.7 ± 6.9 a	4.11 ± 0.32 a
	Met K	68.2 ± 4.6 a	4.33 ± 0.87 a
*T. pityocampa*	Met S	69.8 ± 9.4 a	3.82 ± 0.61 a
	Met K	62.4 ± 12.3 a	4.19 ± 0.48 a

## Data Availability

The data presented in this study are available on request from the corresponding author (S.M.).
